# Update on H5N1 Panzootic: Infected Mammal Species Increase by Almost 50% in Just Over a Year

**DOI:** 10.1111/irv.70159

**Published:** 2025-09-10

**Authors:** Pablo Plaza, Sergio A. Lambertucci

**Affiliations:** ^1^ Grupo de Investigaciones en Biología de la Conservación, Laboratorio Ecotono INIBIOMA, Universidad Nacional del Comahue ‐ CONICET San Carlos de Bariloche Argentina

**Keywords:** H5N1, mammals, mixing vessels, panzootic

1

The current panzootic caused by the highly pathogenic avian influenza virus A(H5N1) (hereafter, H5N1) is having devastating effects on animal and ecosystem health; the virus has spread globally, causing alarming mortalities in a wide range of domestic and wild animals [1]. By early 2024, at least 50 mammal species had been reported infected by H5N1, with massive mortalities in some cases; viral mutations suggest the virus is adapting to infect mammals [2]. This epidemiological situation puts humans at risk due to the potential emergence of a new viral variant capable of triggering a new pandemic. Here, we provide a global update on mammals infected by H5N1 up to July 2025 following the methodology previously used by Plaza et al. [2], which is based on a search of scientific literature and diverse global databases.

## 2 Mammal Species Infected

**FIGURE 1:**
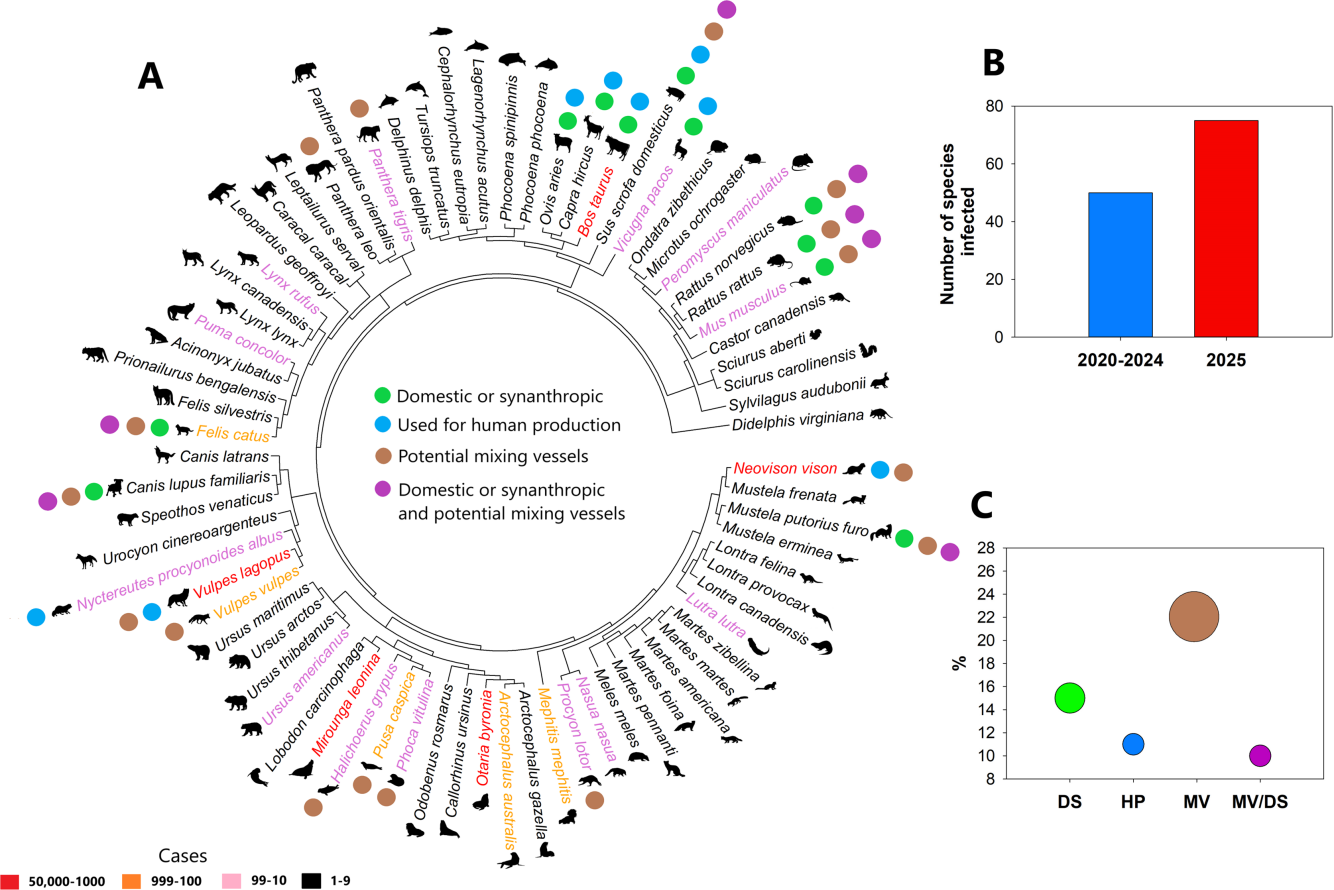
(A) Phylogenetic tree of mammalian species reported as infected by H5N1. The color of the species names represents the number of reported cases in the literature and databases. Colored dots indicate whether a species is domestic or synanthropic, used for human production, a potential mixing vessel, or meets all these criteria. (B) Number of species infected from 2020 to March 2024 and from March 2024 up to July 2025. (C) Percentage of species categorized as domestic or synanthropic, used for human production, or considered potential mixing vessels (bubble size represents the percentage magnitude). DS, domestic or synanthropic; HP, used for human production; MV/DS, domestic or synanthropic and potential mixing vessels; MV, potential mixing vessels. See main text for how the data presented in this figure were obtained.

We found that between March 2024 and July 2025, 24 new species were recorded as infected, representing almost a 50% increase in the number of species (Figure 1A,B). The current 74 mammalian species known to be infected by this virus include domestic, synanthropic (i.e., wild species that live in human‐modified environments and obtain benefits from humans), and wild species (Figure 1A). Humans use some of these species for productive purposes, such as breeding or harvesting for fur and food (Figure 1A), and several may act as mixing vessels (Figure 1A). Even considering the figures are underestimations [1], the Mustelidae family is the most affected (more than 50,000 cases, mostly in captivity for fur production), followed by the families Otariidae (> 24,000 cases, in the wild), Phocidae (> 18,000 cases, in the wild), Canidae (> 11,000 cases, mostly in captivity for fur production), and Bovidae (> 1000 cases, in dairy production) (Figure 1A).

### 2.1 Domestic and Synanthropic Species

Around 1 in 7 of the reported mammalian infected species are domestic (11%, *n* = 8) or synanthropic (4.0%, *n* = 3) (Figure 1A,C); these species live close to humans. The house mouse ( *Mus musculus* ), black rat ( *Rattus rattus* ), Norway rat ( *Rattus norvegicus* ), cow ( *Bos taurus* ), cat ( *Felis catus* ), and dog ( *Canis lupus familiaris* ) have been infected by this virus, with a marked increase in infections since 2024. They usually have large populations and are widespread globally. The proximity of these animals to humans and the large size of their populations present a significant risk of H5N1 spread to human populations everywhere.

### 2.2 Wild Species

This pathogen infected several wild mammal species (85%, *n* = 63) (Figure 1A), some of conservation concern, with serious consequences for their populations [1]. In contrast to synanthropic and domestic species, the spread from wild mammals to humans is expected to pose less risk, as they do not generally live close to humans. Nevertheless, large mortalities—particularly of large‐bodied animals—can attract scavengers (e.g., feral dogs), which may then come into contact with people in urbanized areas, representing a potential risk.

### 2.3 Species Used for Human Production

Among the most impacted mammalian species in terms of number of cases are those used by humans for production purposes (11%, *n* = 8), such as for fur and food (Figure 1A,C). For instance, at least 50,000 minks ( *Neovison vison* ) and thousands of Arctic foxes ( *Vulpes lagopus* ) used for fur production have been infected, resulting in massive mortalities (from either dying or being euthanized) [2, 3, 4]. At least 1020 dairy farms in the United States have tested positive for H5N1 [4]. In some outbreaks (e.g., in minks), mammal‐to‐mammal transmission seems to have occurred [2, 4]. These species pose a significant risk, particularly to workers involved in these production systems.

### 2.4 Mixing Vessel Species

Almost a quarter of the mammalian species infected (approximately 22%, *n* = 16) are considered potential mixing vessels (Figure 1A,C) [5]. Seven of them are also domestic or synanthropic (Figure 1A,C). These species should be considered of concern for public health, as they have large populations, live close to humans, and may spread a virus capable of binding to the human α2,6‐linked sialic acid receptor [5]. Health organizations should closely monitor these species of high risk to humans.

## 3 Concluding Remarks

The number of mammalian species infected by this zoonotic pathogen and the number of cases are rapidly increasing (Figure 1A,B). Although increased testing may have influenced this result, the almost 50% rise in species in just over a year is highly concerning. Some species pose a significant risk to humans due to their large populations, close proximity to human settlements, and potential role as mixing vessels.

Surveillance for high‐risk species (i.e., mixing vessels with large populations living near humans) should be a global priority. The lack of information about the H5N1 epidemiological situation in some regions (e.g., some areas of the Global South) should be addressed by promoting surveillance programs and providing funds and technology [1].

The most affected mammalian species are those used by humans, particularly in intensive production systems. This makes management of the H5N1 spread challenging because the virus is strongly associated with our unsustainable ways of living and production methods [6]. If this panzootic is not addressed from a holistic, ecological, productive, and interdisciplinary perspective, only palliative action will be possible, with a limited effect in reducing the negative impact of this pathogen. H5N1 is already spread globally—not only in birds but also in mammals; it is time to put all our effort into reducing its impacts on wild and domestic species but also its spread to humans.

## Author Contributions

**Pablo Plaza:** conceptualization, data curation, investigation, writing – original draft, writing – review and editing, methodology, funding acquisition. **Sergio A. Lambertucci:** conceptualization, methodology, data curation, investigation, project administration, funding acquisition, writing – original draft, writing – review and editing, supervision.

## Ethics Statement

The authors have nothing to report.

## Consent

The authors have nothing to report.

## Data Availability

The authors have nothing to report.
